# Measured and perceived affordability of asthma medications and their associations with medication adherence among adult patients in Ethiopia: A cross-sectional study

**DOI:** 10.1016/j.rcsop.2026.100832

**Published:** 2026-08-18

**Authors:** Abraham Nigussie Assefa, Fisseha Nigussie Dagnew, Muluken Adela Alemu, Achenef Bogale Kassie, Yohannes Shumet Yimer

**Affiliations:** a Department of Social and Administrative Pharmacy, College of Health Sciences, Debre Tabor University, Debre Tabor, Ethiopia; b Department of Clinical Pharmacy, College of Health Sciences, Debre Tabor University, Debre Tabor, Ethiopia; c Department of Pharmacology, College of Health Sciences, Debre Tabor University, Debre Tabor, Ethiopia

**Keywords:** Asthma, Medication affordability, Perceived affordability, Medication adherence, Health insurance, Ethiopia

## Abstract

**Introduction:**

Effective asthma management depends on regular use of essential medications, yet high costs and low affordability often hinder adherence. This study assessed measured and perceived affordability of asthma medications, examined their associations with medication adherence, and identified factors associated with perceived affordability among patients in the Amhara Region, Ethiopia.

**Methods:**

An institution-based cross-sectional study was conducted from May to August 2024, including 312 eligible asthma patients attending four high-volume hospitals in the region. A total of 296 patients were consecutively sampled during the study period, utilizing structured interviewer administered questionnaires to collect data on socio-demographic, clinical, economic, and perceptions of medication affordability. Validity and reliability of the instruments were established through expert review, factor analysis, and Cronbach's alpha, ensuring accurate measurement of perceived affordability and adherence, with affordability assessed via income-based thresholds and medication adherence measured by the MARS scale. Analysis in SPSS involved descriptive statistics, Spearman's correlation, and multivariable logistic regression to explore relationships and predictors related to affordability and adherence.

**Results:**

Measured affordability was not significantly correlated with perceived affordability (ρ = −0.013, *p* = 0.826) or adherence (ρ = −0.024, *p* = 0.678). In contrast, perceived affordability showed a moderate positive correlation with adherence (ρ = 0.333, *p* < 0.001). Factors significantly associated with higher perceived affordability included health insurance coverage (AOR =2.693, 95% CI:1.202–6.036, *p* = 0.016), being prescribed fewer than two medications (AOR =3.444, 95% CI: 1.777–6.687, *p* < 0.001), medication availability in hospital pharmacy dispensaries (AOR = 5.549, 95% CI: 2.777–11.09, *p* < 0.001), absence of hospitalization (AOR =2.299, 95% CI: 1.15–4.13, *p* = 0.018), and irregular follow-up (AOR =3.413, 95% CI: 1.416–8.23, *p* = 0.006). Conversely, government employment (AOR =0.244, 95% CI: 0.08–0.0.748, *p* = 0.014) and higher travel costs (AOR = 0.986, 95% CI: 0.976–0.997, *p* = 0.01) reduced perceived affordability.

**Conclusion:**

Perceived affordability, rather than measured affordability, significantly influences medication adherence among patients with asthma. Enhancing perceived affordability through expanded health insurance, consistent drug availability, reduced indirect costs, and promoting regular follow-up may improve treatment adherence and asthma outcomes by considering telemedicine, mobile health, or community-based follow-ups to cut transportation costs and save patients time.

## 1 Introduction

Asthma is a heterogeneous disease characterized by chronic airway inflammation, with a history of variable respiratory symptoms such as wheeze, shortness of breath, chest tightness, and cough that vary over time and in intensity, together with variable expiratory airflow limitation.^1^These symptoms may be triggered or exacerbated by factors such as allergens, physical activity, and environmental conditions. Millions of people across different age groups and socioeconomic backgrounds are affected by asthma, resulting in a substantial global health burden.^2^, ^3^Effective asthma management requires continuous pharmacological and non-pharmacological interventions to control symptoms, prevent exacerbations, and improve patients' quality of life.^4^, ^5^ Pharmacological management primarily involves controller medications, particularly inhaled corticosteroids, to reduce airway inflammation and reliever medications such as short-acting beta-agonists to provide rapid symptom relief.^6^, ^7^

Despite the availability of evidence-based treatment guidelines and effective asthma medications, optimal asthma control remains a challenge, particularly in low- and middle-income countries (LMICs). Major barriers include inadequate healthcare infrastructure, limited access to routine follow-up care, poor availability of essential medicines, unaffordability of medications, and suboptimal adherence to prescribed therapies.^8^, ^9^ Medication affordability is a critical component of healthcare access because patients with chronic conditions require continuous and long-term treatment.^10^, ^11^, ^12^High medication costs may lead to delayed treatment initiation, dose reduction, medication discontinuation, and poor disease control, ultimately increasing morbidity and healthcare utilization.^12^, ^13^Therefore, ensuring equitable access to affordable asthma medicines is essential for achieving effective disease management and improving health outcomes. In chronic illnesses such as asthma where continuous management is required to prevent exacerbations the high cost of inhalers and corticosteroids poses a major barrier to adherence, often resulting in missed doses or discontinuation of therapy.^13^, ^14^

Medication availability represents another fundamental dimension of access to asthma care.^15^, ^16^However, persistent stock-outs and unreliable supply of asthma medicines remain common challenges in resource-limited settings.^17^, ^18^Limited availability may force patients to obtain medicines from private pharmacies at higher prices, increase transportation costs due to repeated visits to alternative sources, and contribute to treatment interruptions.^19^ Thus, medication availability and affordability are closely interconnected; supply chain weaknesses may increase the actual financial burden experienced by patients even when medicine prices remain unchanged. Affordability is therefore a multidimensional concept that extends beyond the objective cost of medicines. Measured affordability provides an economic assessment of financial accessibility based on indicators such as medicine prices, income, or healthcare expenditure. However, these objective measures may not fully capture patients' experiences of medication-related financial burden. Perceived affordability reflects patients' subjective assessment of whether they can sustainably obtain and continue their treatment within the context of household priorities, income stability, healthcare financing mechanisms, and competing expenditures. Consequently, medicines considered affordable based on objective measures may still be perceived as unaffordable by patients, potentially influencing medication-taking behaviors and adherence. Similarly, health insurance coverage may improve measured affordability by reducing direct out-of-pocket expenditure, but its influence on perceived affordability may depend on the extent of medicine coverage, availability of insured medicines, and patients' socioeconomic circumstances. Although previous studies have demonstrated associations between medication costs and adherence, evidence remains limited regarding whether perceived affordability provides additional information beyond measured affordability in explaining adherence behaviors, particularly among patients with chronic respiratory diseases in LMICs.

Understanding these distinct but complementary dimensions of affordability is particularly important in Ethiopia, where chronic disease management is challenged by limited resources, medicine supply constraints, and substantial out-of-pocket healthcare expenditures. Although previous studies have examined medication costs, financial barriers, and their associations with asthma medication adherence, limited evidence exists regarding the simultaneous assessment of measured and perceived affordability and their differential relationships with adherence, particularly among patients with asthma in low- and middle-income countries. Therefore, this study aims to examine the association between measured affordability, perceived affordability and medication adherence and identify factors influencing patients' perceptions of affordability among patients with asthma in the Amhara Region, Ethiopia. The findings may provide evidence to inform policy decisions aimed at improving equitable access to asthma medications.

## 2 Conceptual framework

Sociodemographic, clinical, and health system/economic characteristics are hypothesized to be associated with perceived affordability. Measured affordability is hypothesized to be associated with perceived affordability and medication adherence, while perceived affordability is hypothesized to be associated with medication adherence. The framework was developed to guide variable selection and statistical analyses in the present study.

## 3 Methods

### 3.1 Study area

The study was conducted in selected public hospitals in South Gondar Zone, Amhara Regional State, Ethiopia. The zone comprises predominantly rural communities with urban populations served by public healthcare facilities. The included hospitals provide routine asthma diagnosis, follow-up, and medication services and represent healthcare facilities serving diverse patient populations.

### 3.2 Study design

An institution-based cross-sectional study design was employed. Data collection was conducted from May 16 to August 30, 2024.

### 3.3 Source and study population

The source population consisted of adult asthma patients who were receiving outpatient services in the selected hospitals during the study period. The study population included all eligible asthma patients who attended the outpatient clinics and received prescribed medications.

### 3.4 Inclusion criteria

Patients aged 18 years and above who had a confirmed diagnosis of asthma, received prescriptions for asthma medications during their outpatient visit, and who voluntarily consented to participate in the study were included.

### 3.5 Exclusion criteria

Patients who were critically ill at the time of data collection, those unable to communicate effectively, and those with incomplete medical or socioeconomic information were excluded.

### 3.6 Sample size determination

The study included all eligible asthma patients attending in four hospitals during the data collection period. The total estimated population of asthma patients within the study area was approximately 312 individuals. The hospitals were selected based on their high patient volume, ensuring a representative sample of the asthma patient population in the region. During the study period, a total of 296 patients who met the inclusion criteria were approached and enrolled. The sampling was based on consecutive sampling, aiming to include every eligible patient attending the outpatient clinics during this period.

### 3.7 Data collection instruments and procedures

Data were collected using a validated, structured, interviewer-administered questionnaire designed to capture patients' socio-demographic, clinical, economic, and health system characteristics, as well as their perceptions of asthma medication affordability. The section assessing perceived affordability was prepared by reviewing previously published studies.^20^, ^21^, ^22^, ^23^

### 3.8 Development and validation of the perceived affordability questionnaire

The perceived affordability questionnaire was developed through a multistep process. Initially, items were adapted from previously published studies and modified to reflect the Ethiopian context of asthma medication affordability. The draft questionnaire was reviewed by an expert panel comprising two health economics experts, one pharmaceutical supply chain management expert, and two community pharmacists to assess its face and content validity in terms of relevance, clarity, comprehensiveness, and contextual appropriateness. Based on their recommendations, 10 items were retained for further evaluation, with each item measured on a five-point Likert scale ranging from 1 (strongly disagree) to 5 (strongly agree). The questionnaire was originally developed in English, translated into Amharic by bilingual experts, and independently back-translated into English by another bilingual translator to ensure conceptual and linguistic equivalence. Any discrepancies identified during the translation process were resolved through discussion among the research team before finalizing the questionnaire. All face-to-face interviews were conducted in Amharic by trained data collectors.

Subsequently, the questionnaire was pilot-tested among 15 adult asthma patients attending the asthma follow-up clinic at Woldia Comprehensive Specialized Hospital. These participants had characteristics similar to those of the study population but were not included in the final analysis. The pilot test was conducted to assess the clarity, comprehensibility, sequence, and feasibility of the questionnaire. Based on participants' feedback and the pilot findings, minor revisions were made to improve the wording and clarity of several items before the questionnaire was finalized for data collection. Formal cognitive interviews were not conducted.

Construct validity of the 10-item questionnaire was evaluated using exploratory factor analysis (EFA) among 100 participants. The suitability of the data for factor analysis was confirmed by a Kaiser–Meyer–Olkin (KMO) value of 0.923 and a significant Bartlett's test of sphericity (χ^2^ = 508.286, df = 45, *p* < 0.001). Principal component analysis (PCA) with Varimax rotation was initially performed. Based on the eigenvalue >1 criterion, two components were extracted, explaining 64.77% of the total variance. However, because the second component contained only a single item and lacked conceptual interpretability, a one-factor solution was further evaluated in accordance with the theoretical assumption that perceived affordability represents a single underlying construct. Item retention was based on factor loadings (≥0.40), communalities, corrected item-total correlations, and conceptual relevance. During the final validation process, two items were removed. The item *“I can afford my medications without worry”* was excluded because it showed poor correlation with the overall scale, whereas *“I believe pharmaceutical companies price medications fairly”* was removed because of its relatively low communality and limited contribution to the underlying construct. Consequently, the final perceived affordability questionnaire consisted of eight items. The internal consistency of the final eight-item questionnaire was assessed using Cronbach's alpha, which demonstrated good reliability (α = 0.844).

Income estimation was adapted to participants' occupational categories to better reflect the diversity of income sources in the study population. For farmers, total income from the most recent agricultural production season was estimated and converted into an average monthly income. For merchants, average weekly profit was reported and multiplied by four to obtain a monthly estimate. For daily laborers, income was calculated by multiplying the average daily wage by the typical number of working days per month. For housewives and participants without independent income, total household income reported by the household head was used as a proxy for economic capacity.

Measured affordability of asthma medications was assessed using an income-based approach that compared each patient's monthly out-of-pocket expenditure on prescribed asthma medication with their monthly household income. For each participant, the monthly medication cost was divided by the monthly household income and expressed as a percentage to determine the proportion of income spent on asthma treatment. This percentage, referred to as the medication burden, represents the objective level of financial pressure associated with purchasing asthma medication. In this study, a threshold of 10% of household monthly income was used to classify affordability, following catastrophic health expenditure framework. Accordingly, patients whose medication expenditure exceeded 10% of their monthly household were categorized as experiencing unaffordable or catastrophic medication costs, while those spending 10% or less were considered to have affordable medication costs.^24^

Perceived affordability was measured using an eight-item questionnaire, with each item rated on a five-point Likert scale (1 = strongly disagree to 5 = strongly agree). Item scores were summed to obtain a total score ranging from 8 to 40, with higher scores indicating greater perceived affordability. As no validated cutoff was available for this instrument, the scale midpoint (24) was used to categorize the total score. Participants with scores of 25–40 were classified as having high perceived affordability, whereas those with scores of 8–24 were classified as having low perceived affordability. This binary variable was used in the binary logistic regression analysis.

Medication Adherence Reporting Scale (MARS): A 10-item questionnaire used to assess patient adherence to asthma medication.^25^ The instrument has shown strong construct and criterion validity in previous study. For descriptive purposes, participants with MARS scores greater than or equal to the sample mean (3.51) were considered to have relatively higher medication adherence, whereas those with scores below the mean were considered to have relatively lower medication adherence. This categorization was used only to summarize adherence levels and was not used in the correlation analyses, which were performed using the continuous MARS score.

Data were collected by trained research assistants who were health professionals with prior data collection experience. They received two days of training on study objectives, ethical considerations, and interview techniques. Informed consent was obtained from all participants prior to data collection, and participants were assured of their right to withdraw at any time without penalty.

### 3.9 Data analysis

Data were entered into EpiData version 4.6 and exported to SPSS version 26 for statistical analysis. Prior to analysis, data cleaning and coding were performed to check for completeness, consistency, and outliers. Descriptive statistics such as frequencies, percentages were computed to summarize socio-demographic, clinical, health system and economic characteristics of the participants.

To examine the relationships among measured affordability, perceived affordability, and medication adherence, Spearman's rank-order correlation analysis was employed. This non-parametric test was chosen because the data did not meet the assumption of normality, as verified using the Shapiro–Wilk test and visual inspections of histograms and Q–Q plots. Spearman's rho (ρ) was calculated to determine the strength and direction of associations between the variables. Specifically, correlations were computed between (1) measured affordability and perceived affordability, (2) measured affordability and medication adherence, and (3) perceived affordability and medication adherence. A two-tailed *p*-value of less than 0.05 was considered statistically significant for all analyses. The assumptions and performance of the binary logistic regression model were assessed before interpretation of the regression estimates. Multicollinearity among independent variables was evaluated using variance inflation factor (VIF) and tolerance values, with VIF <5 and tolerance >0.20 considered acceptable. Model calibration was assessed using the Hosmer–Lemeshow goodness-of-fit test. Classification performance was evaluated using the overall percentage of correctly classified cases, sensitivity, and specificity from the classification table. Model discrimination was assessed using the receiver operating characteristic (ROC) curve and the area under the curve (AUC). Potential influential observations were evaluated using standardized residuals, Cook's distance, and leverage values. Bivariate analysis was first performed to identify variables associated with perceived affordability, including socio-demographic, clinical, health system and economic factors. Variables with a *p*-value <0.2 in bivariate analysis were entered into a multivariable binary regression model to determine independent predictors. A *p*-value <0.05 was considered statistically significant.

### 3.10 Ethical considerations

Ethical approval for this study was obtained from the Institutional Review Board (IRB) of the College of Health Sciences, Debre Tabor University Reference number DTU/RES/446/24. Following IRB approval, a support letter was issued by the University, and formal administrative permission to conduct the study was obtained from the administrations of all participating public hospitals before participant recruitment and data collection. Written informed consent was obtained from all participants after explaining the purpose of the study, and participation was voluntary. Participant confidentiality and privacy were maintained throughout the study by using anonymized data, omitting personal identifiers, and restricting access to the collected information to the research team. All study procedures were conducted in accordance with the ethical principles of the Declaration of Helsinki.

## 4 Result

### 4.1 Model diagnostic result

**Fig. 1:**
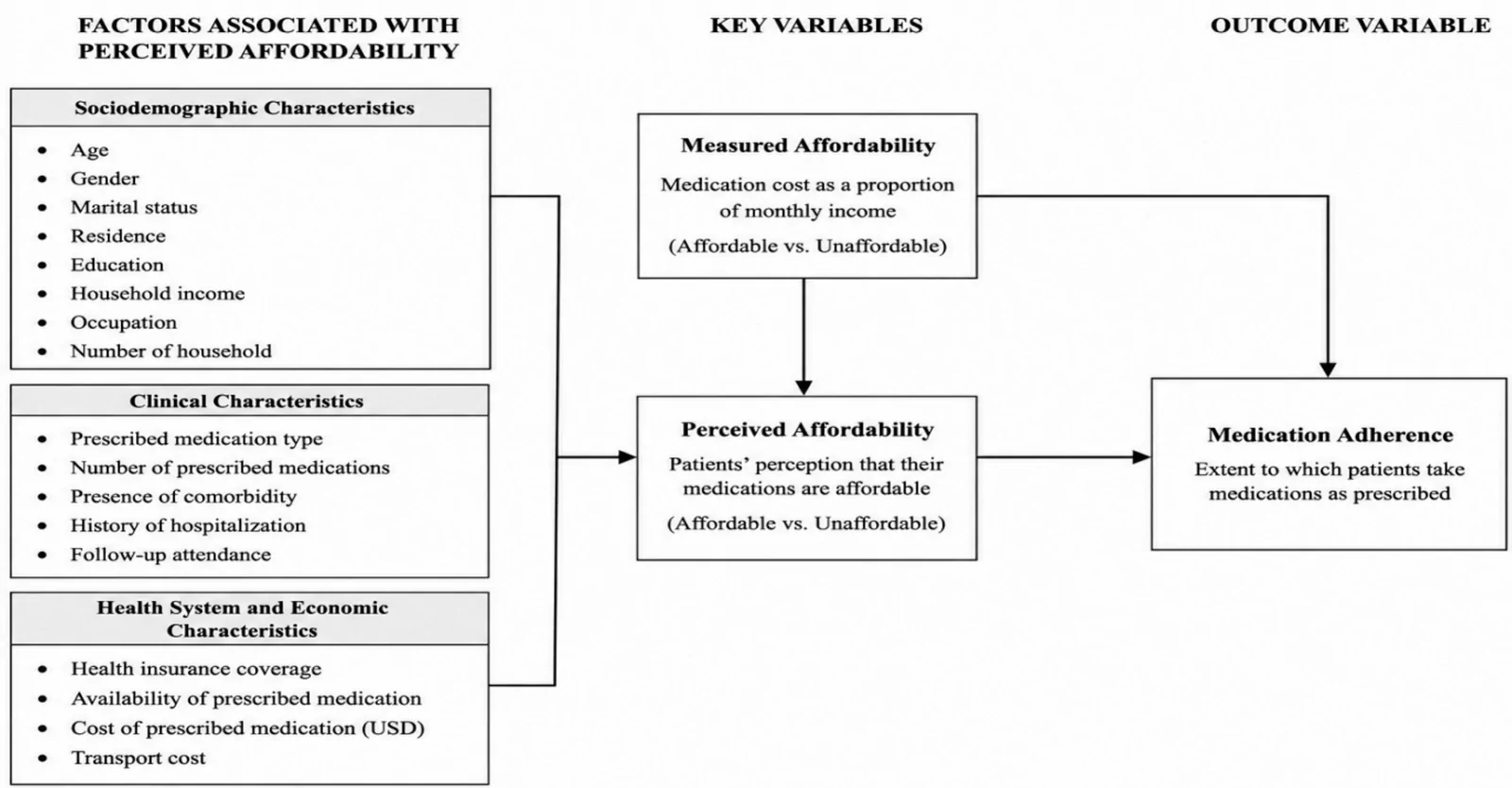
Conceptual framework illustrating the hypothesized associations among measured affordability, perceived affordability, medication adherence, and factors associated with perceived affordability among patients with asthma in the Amhara Region, Ethiopia.

The binary logistic regression model satisfied the diagnostic assumptions. No evidence of the logistic regression model satisfied the diagnostic assumptions. VIF values ranged from 1.085 to 3.504 and tolerance values ranged from 0.285 to 0.922, indicating no evidence of problematic multicollinearity. The Hosmer–Lemeshow goodness-of-fit test indicated an adequate model fit (*p* = 0.817). The model correctly classified 76.0**%** of participants, with a sensitivity of 75.3% and specificity of **7**6**.7%**. The model also demonstrated good discriminative ability (AUC = 0.857). Standardized residuals, Cook's distance, and leverage values indicated that no influential observations substantially affected the model estimates (Table 1). (See Fig. 1.)

**Table 1:** Diagnostic assessment and performance of the final binary logistic regression model.

Assessment	Statistic	Result
Multicollinearity	VIF	1.085–3.504
	Tolerance	0.285–0.922
Model goodness-of-fit	Hosmer–Lemeshow test	*p* = 0.817
Classification performance	Overall classification accuracy	76.0%
	Sensitivity	75.3%
	Specificity	76.7%
Model discrimination	ROC (AUC)	0.857(85.7%)
Influential observations	Standardized residuals	−2.263 to 2.490
	Cook's distance	0.00009–0.422
	Leverage values	0.006–0.187

### 4.2 Sociodemographic and socioeconomic characteristics of respondents

A total of 296 asthma patients were approached for participation, and all completed the questionnaire, resulting in a 100% response rate. No eligible participants declined participation, and no questionnaires were excluded due to incomplete responses. The sociodemographic characteristics of the participants are presented in Table 2. The mean age of respondents was 42 years (SD = ±9.5). The gender distribution was relatively balanced, with 55.1% males and 44.9% females. The majority of participants was married (59.8%) and resided in urban areas (81.1%). Regarding educational status, 30.7% had completed higher education, 29.7% had secondary education, and 9.8% were illiterate. Most participants reported a monthly household income ranging from 6500 to 7900 ETB, accounting for 61.5% of respondents. Regarding occupation, 31.8% were merchants, 30.4% were government employees, 15.9% were farmers, 14.2% were daily laborers, and 7.8% were housewives. More than half of the respondents (56.1%) lived in households contained 3–4 members.

**Table 2:** Sociodemographic Characteristics of Adult Asthma Patients Attending Public Hospitals in the South Gondar Zone, Amhara Region, Ethiopia (*n* = 296).⁎

Variable	Category	n (%)
Age (years)	Mean ± SD	42 ± 9.5
Gender	Male	163 (55.1)
	Female	133 (44.9)
Marital Status	Married	177 (59.8)
	Divorced	103 (34.8)
	Single	16 (5.4)
Residence	Urban	240 (81.1)
	Rural	56 (18.9)
Education	Illiterate	29 (9.8)
	Primary school	88 (29.7)
	Secondary school	88 (29.7)
	Higher education	91 (30.7)
Household Income (ETB)	3500–5000	12 (4.1)
	5001–6500	75 (25.3)
	6501–7900	182 (61.5)
	>7901	27 (9.1)
Occupation	Government employee	90 (30.4)
	Farmer	47 (15.9)
	Housewife	23 (7.8)
	Merchant	94 (31.8)
	Daily laborer	42 (14.2)
Number of household	1–2	57(19.3)
	3–4	166 (56.1)
	>5	73(24.7)

⁎ 1 USD = 122.70 ETB on the day 30/10/2024.

### 4.3 Clinical and disease-related characteristics

The majority of participants (74.3%) were prescribed a combination of controller and reliever medications, while 19.3% used controller medications alone and 6.4% used reliever medications only. Most participants (60.8%) were prescribed more than two medications. Nearly half (49%) reported having at least one comorbidity. More than one-third (36.1%) of patients had a history of hospitalization. Regarding follow-up attendance, 41.9% attended sometimes and 20.6% of respondents were attending regularly (Table 3).

**Table 3:** Clinical and Disease-Related Characteristics of Adult Asthma Patients Attending Public Hospitals in the South Gondar Zone, Amhara Region, Ethiopia (*n* = 296).

Variable	Catagory	N(%)
Prescribed medication type		
	Controller	57(19.3)
	Reliever	19(6.4)
	Combination	220(74.3)
The number of prescribed medications		
	≤2	116(39.2)
	>2	180(60.8)
Presence of comorbidity		
	Yes	145(49)
	No	151(51)
History of hospitalization		
	Yes	107(36.1)
	No	189(63.9)
Follow-up attendance		
	Regular	61(20.6)
	Sometimes	124(41.9)
	Irregular	111(37.5)

### 4.4 Health system and economic characteristics

A considerable proportion (61.8%) had health insurance coverage. From totally prescribed asthma medication, 46% get there medication within health facilities under the study. The cost of prescribed medications varied, with 48.3% of participants spending between 200 and 750 ETB per prescription. Regarding transportation expenses, slightly more than half (51.7%) spent more than 50ETB including expenses to both the health facility and pharmacy (Table 4).

**Table 4:** Health System and Economic related Characteristics of Adult Asthma Patients Attending Public Hospitals in the South Gondar Zone, Amhara Region, Ethiopia (*n* = 296).⁎

Variables	Category	N (%)
Health insurance coverage		
	Yes	183(61.8)
	No	113(38.2)
Availability of prescribed medication		
	Yes	136(46)
	No	160(54)
Cost of prescribed medication(ETB)		
	200–750	124 (41.9)
	751–1250	143 (48.3)
	>1251	29 (9.8)
Transport cost(including cost to Pharmacy and Health facility (ETB)		
	<50	143(48.3)
	≥50	153(51.7)

⁎ 1 USD = 122.70 ETB on the day 30/10/2024.

### 4.5 Descriptive analysis of affordability and medication adherence scores

The mean measured affordability score was 12.25 ± 5.45, with a median of 11.0 (IQR = 7.0) and a range of 3–36. The mean perceived medication affordability score was 2.50 ± 0.38, with a median of 2.50 (IQR = 0.50) and a range of 1.25–3.38. The medication adherence (MARS) score had a mean of 35.15 ± 7.00, a median of 36.0 (IQR = 10.75), and ranged from 18 to 50(Table 5).

**Table 5:** Distribution of perceived medication affordability scores among patients with asthma (*N* = 296).

Variable	Mean ± SD	Median (IQR)	Minimum–Maximum
Measured affordability score	12.25 ± 5.45	11.0 (7.0)	3–36
Perceived affordability score (mean item score)	2.50 ± 0.38	2.50 (0.50)	1.25–3.38
Medication adherence (MARS) score	35.15 ± 7.00	36.0 (10.75)	18–50

### 4.6 Measured and perceived affordability of prescribed asthma medications

**Fig. 2:**
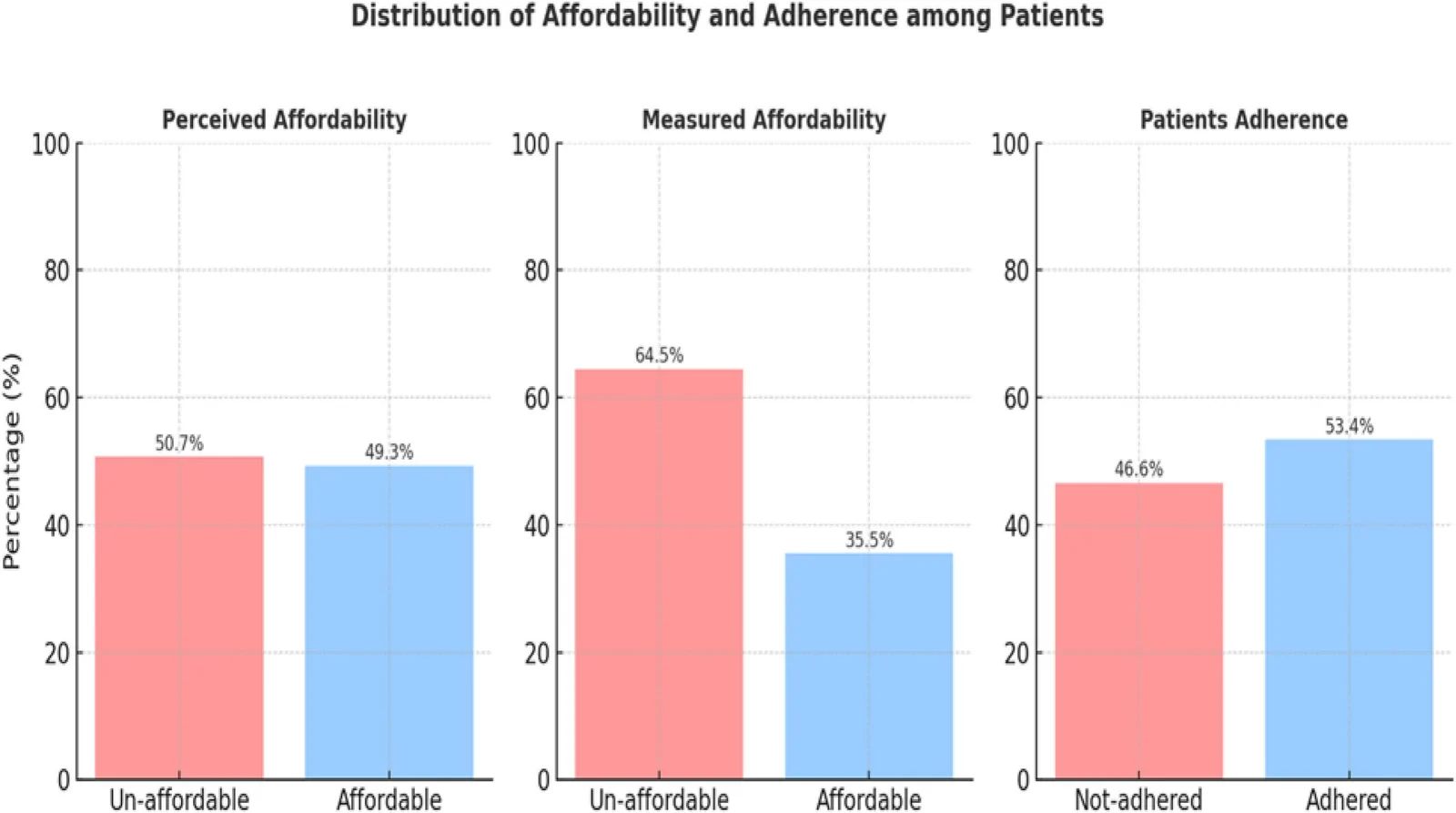
Perceived and measured affordability of prescribed asthma medications with adherence of patients for medication (*n* = 296).

Fig. 2 illustrates the distribution of measured affordability, perceived affordability, and medication adherence among patients with asthma. Among the 296 participants, 105 (35.5%) were classified as having affordable asthma medications based on the objective affordability assessment, whereas 146 (49.3%) perceived their medications to be affordable. Therefore, perceived affordability was higher than objectively measured affordability, suggesting that some participants considered their medications affordable despite the objective assessment indicating otherwise. Medication adherence for prescribed medication 53.4% of patients were adherent to medication and the other were not adhered to medication.

### 4.7 Relationships between measured affordability, perceived affordability, and

**Fig. 3:**
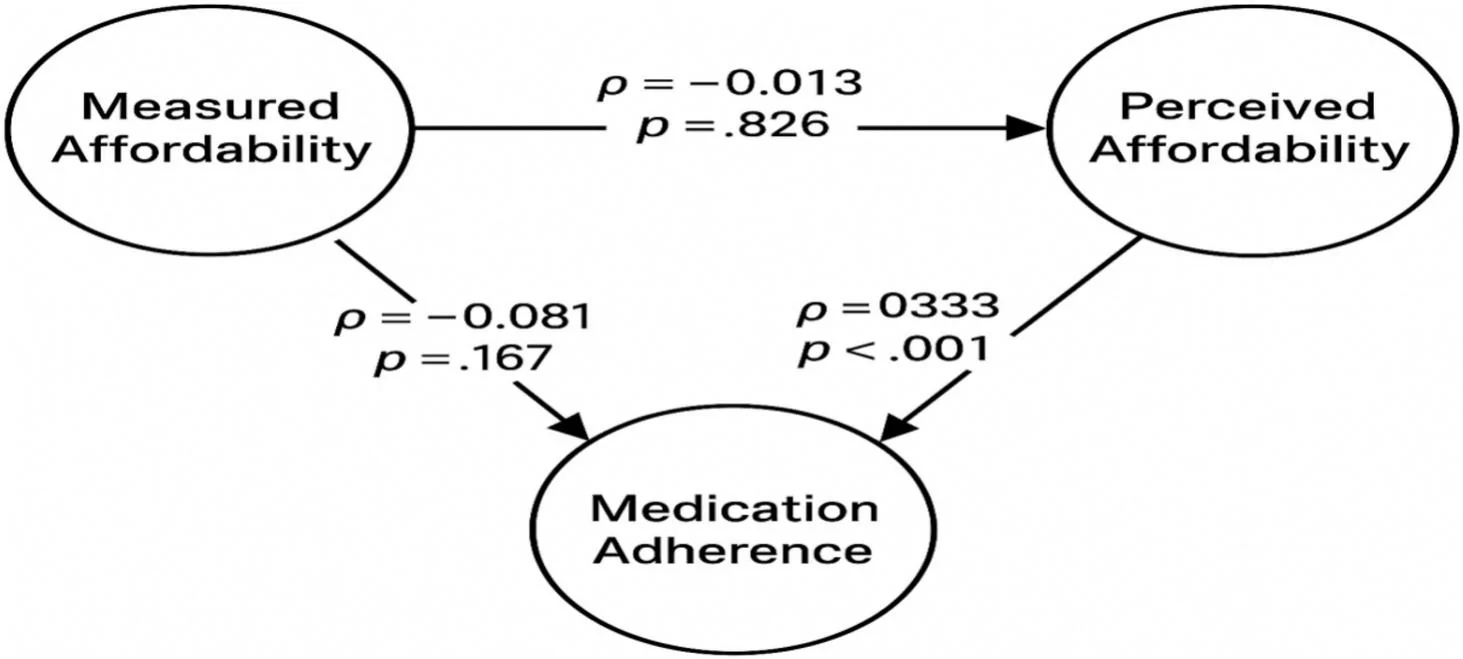
Correlations between Measured Affordability, Perceived Affordability, and Medication Adherence (*N* = 296).

Correlation analysis revealed a moderate positive correlation between perceived affordability and medication adherence (Spearman's ρ = 0.333, *p* < 0.001), indicating that patients who perceived their medications as more affordable tended to report better adherence. In contrast, measured affordability showed essentially no correlation with medication adherence (Spearman's ρ = −0.013, *p* = 0.824), suggesting no meaningful association between the objective affordability measure and adherence in this study (Fig. 3).

### 4.8 Factors affecting perceived affordability

Binary logistic regression was performed to identify predictors of perceived affordability of asthma medications among patients attending outpatient clinics. As presented in Table 4, several variables were significantly associated with perceived affordability (*p* < 0.05).Occupation type also showed a significant overall association (*p* < 0.001), with government-employed patients being less likely to perceive medications as affordable (AOR =0.244, 95% CI: 0.08–0.748, *p* = 0.014).Patients with health insurance coverage were 2.7 times more likely to perceive their asthma medications as affordable compared to those without insurance (AOR =2.693, 95% CI:1.202–6.036, *p* = 0.016). Similarly, those who reported that prescribed medications were available in the hospital pharmacy dispensary had a 5-fold higher likelihood of perceiving affordability (AOR =5.549, 95% CI: 2.777–11.09, *p* < 0.001). Patients who received fewer than two medications were 3.44 times more likely to perceive medication as affordable compared to those who received more than two medications (AOR =3.444, 95% CI: 1.777–6.687, *p <* 0.001).Patients who had no history of hospitalization during their treatment were also more likely to perceive their medications as affordable (AOR =2.299, 95% CI: 1.15–4.13, *p* = 0.018). Conversely, increased travel cost to hospitals or pharmacies was associated with a slight decrease in perceived affordability (AOR = 0.986, 95% CI: 0.976–0.997, *p* = 0.01).Furthermore, patients with irregular follow-up visits demonstrated a 3.4-fold higher likelihood of perceiving affordability compared to those without follow-up (AOR =3.413, 95% CI: 1.416–8.23, *p* = 0.006) (Table 6).

**Table 6:** Multivariate logistic regression analysis of factors affecting patients perceived affordability of asthma medication.

Predictor	*P*-value	AOR	CI
Occupation			
Government employer	0.014	0.244	(0.08,0.748)
Farmer	0.21	2.00	(0.676,5.92)
House wife	0.71	1.254	(0.676,4.284)
Merchant	0.19	1.858	(0.735,4.698)
Daily labor	Reference		
Health insurance coverage			
Yes	0.016	2.693	(1.202,6.036)
No	Reference		
Availability of Prescribed Medication			
Yes	<0.001	5.549	(2.777,11.09)
No	Reference		
Being prescribed fewer than two medications			
≤2	<0.001	3.444	(1.777,6.687)
>2	Reference		
History of Hospitalization			
No	0.015	2.299	(1.179,4.482)
Yes	Reference		
Frequency of Follow up			
Irregular	0.006	3.413	(1.416,8.23)
Sometimes	0.055	2.009	(0.985,4.097)
Regular	Reference		
Travel cost to Hospital or Pharmacy (ETB)	0.01	0.986	(0.976,0.997)

## 5 Discussion

The present study examined both the measured and perceived affordability of asthma medications and their association with patients' adherence to prescribed medications among 296 participants. Objectively, 35.5% of patients were categorized as having affordable medication expenditures, whereas 64.5% encountered unaffordable expenses. The evidence indicates that most asthma patients suffer a significant financial hardship in acquiring their recommended medications. When affordability was assessed subjectively, based on patients' perceptions, 49.3% regarded the medications as affordable, whereas 50.7% considered them unaffordable. This disparity between perceived and measured affordability suggests that patients' opinions don not always coincide with the objective economic assessment of the burden of cost of prescription medications. Depending on personal factors including social support, coping mechanisms, economic stability, or knowledge of the cost of medications, some patients might underestimate or overstate affordability.

Among the participants, 53.4% adhered to their prescribed asthma medications, while 46.6% were non-adherent, indicating a moderate level of adherence and a continuing challenge in asthma management. Spearman's correlation analysis showed a very weak, negative, and non-significant relationship between measured and perceived affordability (ρ = −0.013, *p* = 0.826), indicating that no evidence of a monotonic association was observed between the two measures in this sample. Likewise, measured affordability was not significantly associated with medication adherence (ρ = −0.024, *p* = 0.678), whereas perceived affordability showed a moderate positive correlation with adherence (ρ = 0.333, *p* < 0.001). This finding suggests that patients' perceptions of medication affordability may be more closely associated with adherence than objective affordability measures.^26^The difference between measured and perceived affordability suggests that they capture different but complementary aspects of financial access to medicines. While measured affordability reflects medication costs relative to household income, perceived affordability also incorporates broader financial and contextual factors such as financial security, competing household expenses, transportation costs, family support, coping strategies, previous healthcare experiences, health literacy, and willingness to pay. These factors may partly explain why perceived affordability was significantly associated with medication adherence.

However, this finding should be interpreted with caution. The absence of a statistically significant correlation between measured and perceived affordability does not indicate that the two measures are independent; rather, it suggests that no evidence of a monotonic association was observed in this sample. The result may also reflect limited statistical power, measurement limitations, and possible misclassification arising from objective affordability thresholds and self-reported perceptions. Future studies with larger samples and longitudinal designs are needed to better understand the relationship between objective and perceived affordability.

The stronger association between perceived affordability and medication adherence is consistent with behavioral economics and the Health Belief Model, which suggest that patients' treatment decisions are influenced more by perceived barriers than by objective costs alone. The principal contribution of this study is the finding that perceived affordability was more strongly associated with medication adherence than objectively measured affordability, highlighting the importance of considering both objective and patient-reported affordability when designing interventions to improve medication adherence and access to medicines.

Our study identified several key factors significantly influence patients' perceptions of medication affordability. Multivariate logistic regression analysis revealed that patients with insurance coverage were approximately two times more likely to perceive their medications as affordable compared to patients without insurance. This may be because the absence of insurance coverage substantially increases the financial burden of medication costs this can be reduced by transitioning patients from out-of-pocket payments to prepayment mechanisms represents an important step toward mitigating financial hardship and may improving perceptions of medication affordability.^27^ According to a similar study conducted in Ethiopia, insurance coverage may contributing to perceived access to pharmaceuticals by lowering out-of-pocket costs, acting as a protective factor.^28^ Patients with insurance are more likely to perceive medications as affordable since their coverage alleviates immediate cost strain, a trend observed in multiple settings worldwide.^28^, ^29^ This underscores the importance of expanding insurance coverage to improve perceptions of medication affordability.

Patients who faced medication shortages were almost five times more likely to believe that prescribed medicines were unaffordable. This significant association emphasizes the importance of medication availability in shaping patients' perceptions of their financial situation. Prescription medicine shortages are common in many low- and middle-income countries. A study in Kenya showed that patients' perceptions of unaffordability were strongly influenced by medication unavailability, as they often had to purchase more expensive alternatives.^30^Similarly, other studies have reported that medication shortages increase perceived costs by forcing patients to seek alternatives or postpone treatment, exacerbating affordability problems.^31^, ^32^ Patients in Uganda indicated that repeated trips to facilities, where stock-outs were often discovered, caused financial and emotional burdens, making medications appear costly.^24^ Even insured patients in Ghana experience shortages due to uneven National Health Interview Survey (NHIS) reimbursement and inadequate supply chains, contributing to perceived unaffordability.^33^ In such cases, patients are compelled to purchase medications from private pharmacies or unofficial marketplaces, where costs are typically higher. Some research suggests that unavailable medicines may lead patients to use alternative, sometimes more accessible or less expensive therapies, which can partially alleviate perceived unaffordability.^31^ However, the safety and efficacy of these alternatives may vary, highlighting the importance of a consistent medicine supply for both economic and health reasons.

The relationship between polypharmacy and medication affordability is complex. While our study found that patients prescribed fewer than two medications were significantly more likely to perceive their medications as affordable compared to those prescribed more than two medications, this does not imply that reducing medications arbitrarily is always appropriate. Clinical necessity should always guide prescribing decisions. Instead, optimizing medication regimens to avoid unnecessary polypharmacy may help reduce the financial burden on patients. This approach involves carefully evaluating the need for each medication and discontinuing those that are unnecessary or redundant. Our findings align with previous studies; for example, a study in Jimma Zone, Ethiopia, reported that patients with more than two medications per prescription were less likely to consider their medications affordable.^34^ Additionally, a population-based study on polypharmacy demonstrated that higher numbers of medications are associated with increased medication costs and financial strain.^35^ These evidence points support the idea that rationalizing medication regimens by avoiding unnecessary polypharmacy may improve patients' perceived affordability, but such strategies should always prioritize clinical appropriateness.

In this study, patients without a history of hospitalization were significantly more likely to perceive their medications as affordable compared to those who had been hospitalized. While our study did not directly measure specific costs such as bed charges, it is well-recognized that hospitalization often involves substantial direct costs. This suggests that hospitalization, as a major health event, can impose substantial financial strain on patients and negatively affect their perception of affordability. Hospital stays often involve direct costs, such as bed charges, procedures, medications, and laboratory tests, as well as indirect costs, including transportation, loss of income, and caregiver support. These additional expenses may amplify the perceived burden of ongoing medication costs even after discharge. Patients without a history of hospitalization were more likely to perceive their medications as affordable. This aligns with evidence indicating that hospitalization increases the overall cost of illness and reduces affordability perception. A study in Ethiopia has shown that patients who had been hospitalized incurred higher out-of-pocket expenses for conditions such as hypertension, leading to decreased affordability.^36^ These findings suggest that hospitalization by increasing healthcare costs can negatively influence patients' perception of medication affordability.

In this study, patients who attended regular follow-up visits were less likely to perceive their medications more affordable than those with irregular follow-up. Although this finding appears counterintuitive, it may reflect the broader economic and healthcare context of the Amhara region rather than a true reduction in medication affordability. Regular follow-up for chronic conditions such as asthma requires repeated healthcare visits, resulting in both direct costs (transportation and medication expenses) and indirect costs (lost income and time away from work or household responsibilities). In settings characterized by limited financial resources, these cumulative costs may increase patients' overall financial burden and negatively influence their perception of medication affordability. This finding is consistent with evidence from resource-limited settings, where repeated healthcare utilization has been associated with increased out-of-pocket expenditures among patients with chronic diseases.^18^, ^36^ In the Amhara region, the ongoing socioeconomic challenges, including inflation, conflict-related disruptions, population displacement, and interruptions in healthcare services, may further amplify these financial pressures. Patients attending regular follow-up may also have more severe disease or require more intensive treatment, leading to greater healthcare utilization and higher cumulative expenditures, which could reduce their perceived affordability. Conversely, patients with irregular follow-up may purchase medications less frequently or delay healthcare visits, resulting in lower immediate out-of-pocket spending and a greater perception of affordability. However, this should not be interpreted as indicating that irregular follow-up improves affordability or represents optimal disease management. Given the cross-sectional design of this study, causal relationships cannot be established, and future longitudinal studies are needed to better understand the relationship between follow-up patterns, healthcare costs, and perceived medication affordability.

In this study, higher travel costs to reach hospitals or pharmacies were associated with lower perceived affordability of medications. Although transportation costs were not included in the objective affordability measure, which was based solely on direct medication expenditure relative to monthly income, they may substantially influence patients' perceptions of affordability. This finding reflects the reality in the Amhara region, where conflict, displacement, and infrastructure disruption have increased both the cost and difficulty of accessing healthcare facilities. Many patients must travel long distances because nearby health facilities are damaged, inaccessible, or only partially functional, incurring additional expenses for transportation, food, and, in some cases, accommodation. These indirect costs increase the overall financial burden of obtaining treatment and may contribute to the discrepancy between objectively measured affordability and patients' perceived affordability, as individuals consider the total cost of accessing care rather than medication costs alone.

The findings of this study have important implications for clinical practice and health policy. Pharmacists should routinely assess patients' financial barriers to obtaining medications, provide counseling on cost-effective treatment options, and support medication adherence through patient-centered pharmaceutical care. Physicians should consider patients' financial circumstances when prescribing long-term asthma therapies and, where clinically appropriate, select equally effective but more affordable treatment options. Hospital pharmacy services should strengthen medicine inventory management to minimize stock-outs and ensure the continuous availability of essential asthma medications. At the health system level, strengthening pharmaceutical supply systems is essential to improve medicine availability and reduce patients' reliance on higher-cost private pharmacies. Furthermore, policymakers should prioritize expanding health insurance coverage and implementing financing and procurement strategies that reduce out-of-pocket expenditure for essential medicines. Together, these measures could improve both the objective affordability of asthma medications and patients' perceived ability to access and adhere to treatment.

## 6 Conclusion

Perceived affordability was moderately and significantly associated with better medication adherence, highlighting the importance of patients' financial perceptions in sustaining treatment. In contrast, measured affordability showed no statistically significant association with medication adherence in this study, suggesting that objective cost measures may not adequately capture all factors influencing adherence behavior. Multiple factors significantly influenced perceived affordability of asthma medications, including occupation, insurance coverage, medication availability, number of prescribed medications, hospitalization history, travel costs, and follow-up frequency. Patients with health insurance, fewer medications, and regular drug availability were more likely to perceive their treatment as affordable, whereas higher travel costs and previous hospitalization were associated with lower perceived affordability.

## 7 Recommendation

It is recommended that policymakers and healthcare providers strengthen financial protection mechanisms, such as expanding health insurance coverage and implementing cost-sharing subsidies, to enhance patients' perceived affordability and adherence. Additionally, improving medicine availability at public facilities, minimizing travel-related costs through decentralized services, and optimizing prescription practices to reduce unnecessary poly-pharmacy can further improve patients' access to affordable asthma treatment and promote better health outcomes. Make it patients to understand the importance of regular follow-ups on preventing complications and reducing hospitalizations and increase convenient appointment times or tele-health options to reduce indirect costs like transportation and time off work, thereby improving the perceived value of follow-up care that follow-ups are affordable.

## 8 Strength and limitation of study

### 8.1 Strengths

This study provides a comprehensive assessment of both the measured and perceived affordability of prescribed asthma medications, offering valuable insights into the financial challenges faced by patients in South Gondar Zone hospitals. The use of objective measures, such as the catastrophic payment method, alongside patient-reported perceptions, allowed for a nuanced understanding of affordability gaps. The relatively large sample of 296 patients and consecutive recruitment improved the representativeness of patients attending the selected public hospitals in the study area. The study also examined multiple contributors of perceived affordability providing actionable evidence for policymakers and healthcare providers.

### 8.2 Limitations

Despite these strengths, the study has several limitations. First, the cross-sectional design captures associations but cannot establish temporal or causal relationships between affordability and medication adherence or between the identified determinants and perceived affordability. Second, medication adherence was assessed using the self-reported Medication Adherence Rating Scale (MARS), which may be subject to recall and social desirability biases and could therefore result in over- or underestimation of adherence. Similarly, household income and out-of-pocket expenses were self-reported and may have been underestimated or overestimated, potentially affecting the accuracy of the measured affordability estimates. Third, the objective affordability assessment was based on a 10% threshold of monthly income, which may not fully reflect household financial capacity because it does not account for household size, essential household expenditures, or other competing financial obligations. Fourth, medication availability was assessed based on pharmacy stock on the day of the visit and may not reflect longer-term patterns of medicine availability or stock-outs. Fifth, although several socioeconomic and health-related factors were assessed, residual confounding cannot be excluded because potentially relevant factors such as asthma severity, health literacy, family financial or social support, psychological factors, and informal payments were not fully captured. Finally, the study was conducted among outpatients attending selected public hospitals in the South Gondar Zone, which may limit the applicability of the findings to patients receiving care in private facilities, other geographic areas, or different healthcare settings.

### 8.3 Future research

Future research should employ longitudinal or prospective study designs to better establish the temporal and causal relationships between medication affordability, patients' perceptions of affordability, and medication adherence. Qualitative or mixed-methods studies are also needed to explore how patients form perceptions of medication affordability and how indirect lost income costs, such as transportation, and time spent accessing healthcare, influence these perceptions. In addition, future studies should evaluate interventions aimed at improving medicine affordability and access, including health insurance expansion, strategies to strengthen pharmaceutical supply systems and reduce medicine stock-outs, and pharmacist-led adherence support programs. Multi-center studies involving diverse geographic regions would also enhance the generalizability of findings and provide a broader understanding of affordability challenges across different healthcare settings.

## CRediT authorship contribution statement

**Abraham Nigussie Assefa:** Writing – review & editing, Writing – original draft, Validation, Supervision, Methodology, Investigation, Formal analysis, Data curation, Conceptualization. **Fisseha Nigussie Dagnew:** Writing – review & editing, Visualization, Resources, Investigation, Formal analysis. **Muluken Adela Alemu:** Writing – review & editing, Visualization, Supervision, Investigation, Formal analysis, Data curation. **Achenef Bogale Kassie:** Writing – original draft, Visualization, Resources, Investigation, Formal analysis. **Yohannes Shumet Yimer:** Writing – original draft, Validation, Methodology, Investigation, Data curation, Conceptualization.

## Consent for publication

Not applicable.

## Ethics approval and consent to participate

Ethical clearance for this study was obtained from the Institutional Review Board of Debre Tabor University College of Health Sciences with reference number DTU/RES/446/24. Following ethical approval from the Institutional Review Board, administrative permission to conduct the study was obtained from each participating hospital before participant recruitment and data collection. Participants were informed about the purpose and design of the study before data collection. Additionally, they gave their formal agreement to take part in the study. All study participants provided written informed consent. During the data collection and entry procedure, new codes were substituted for the patients' medical registration numbers (MRNs). Additionally, the collected data was similarly stored on a computer with a strong password and in a locked cabinet to ensure its confidentiality. The study was conducted in accordance with the principles of the Declaration of Helsinki.

## Funding

The author(s) declare that no financial support was received for the research, authorship, and/or publication of this article.

## Declaration of competing interest

The authors declare that they have no known competing financial interests or personal relationships that could have appeared to influence the work reported in this paper.

## Data Availability

Data is provided within the manuscript or supplementary information files. If the additional raw data is needed it will be made available by the reasonable request of the corresponding author (Abraham Nigussie Assefa).
